# The Role of Viruses in Pulpal and Apical Disease: A Systematic Review

**DOI:** 10.3390/v16101537

**Published:** 2024-09-28

**Authors:** Katia Hermosilla Hermosilla, Paula Soto Cárdenas, Manuel Donoso Zuñiga, Cecilia Pérez Ñanco, Scarlette Hernández-Vigueras

**Affiliations:** 1 Dental School, Faculty of Medicine, Austral University of Chile, Valdivia 5090000, Chile; 2 Odontostomatology Institute, Faculty of Medicine, Austral University of Chile, Valdivia 5090000, Chile

**Keywords:** viruses, endodontic, apical diseases, periapical lesions

## Abstract

Apical lesions are diseases of infectious origin that can cause destruction of the surrounding periapical tissue, including bone tissue and periodontal ligaments, resulting in the loss of the affected teeth. Currently, the microorganisms present in pulp and apical disease are mostly studied as bacteria. However, in recent years, interest has been aroused in the study of viruses that could be present in apical lesions, and how these could affect the progression of disease. In the present study, we conducted a systematic review of the literature to evaluate and synthesize the scientific evidence on the presence of viruses and their possible role in pulpal and apical disease. This systematic review was performed according to the PRISMA reporting guidelines. The search for studies was performed in the PubMed and Web of Science databases. A total of seven studies published in the last 10 years were included. The types of samples used for virus analysis varied from one study to another. In all the included studies, the presence of any of the types of viruses studied was found, either in pulp or apical tissue. Herpesviridae family, Epstein–Barr virus (EBV) and Human cytomegalovirus (HCMV) stood out as the most commonly present in apical lesions. Further studies are required to clarify and understand the pathogenic role of viruses in pulpal and periapical disease.

## 1. Introduction

Apical lesions are infectious diseases occurring in the periapical region of the teeth which may cause swelling, pain, or other symptoms. Their development and progression are determined by the interaction of pathogens with the host, specifically with the immune system. If left untreated, they can cause bone tissue resorption, destruction of the periodontal ligament and loss of the affected teeth [1].

Progression to chronic lesions develops as a result of the host’s inability to eliminate the infection. Histologically, these lesions are characterized by fibrous and granulomatous tissue, proliferative epithelium or cysts infiltrated by various inflammatory cells [2].

Historically, the focus of research on microorganisms involved in pulpal and apical diseases has primarily been on bacteria. However, recent studies have highlighted the potential role of viruses in these conditions, sparking renewed interest and investigation into their pathogenic mechanisms. In recent decades, interest in the study of the role of viruses in apical diseases has increased, especially with the introduction of new detection tools such as polymerase chain reaction (PCR)-based amplification and immunohistochemistry which provide a boost in the identification of viral agents [3].

Viruses apply immunoevasive strategies to suppress the antiviral responses of the host, resulting in the presence of different viral diseases as products of cell destruction or secondary to immune responses [3].

In dental practice, it is common to find viral diseases affecting the oral mucosa and the perioral region as some viral agents are important in the appearance of ulcerative lesions, tumors, and periodontal disease [3].

Current investigations of these viral agents are mainly focused on Epstein–Barr (EBV) and Human cytomegalovirus (HCMV), both of the herpesvirus family. It is known that these viruses can persist in a lifelong latency after primary infection, where their most frequent sites of reactivation are the salivary glands and mucosal surfaces [4].

EBV infects oronasopharyngeal epithelial cells in a lytic manner before establishing type I latency in B lymphocytes, while HCMV maintains latency in CD34+ bone marrow progenitor cells, dendritic cell precursors and monocytes [5].

These latent viruses can be reactivated during periapical inflammation (1). EBV reactivation can induce the production of TNF-a, a key mediator of inflammation and tissue destruction, while HCMV reactivation occurs during differentiation of infected monocytes into macrophages or maturation of dendritic cells, which facilitates the spread of infectious virions to additional macrophages, T lymphocytes, endothelial cells and connective tissue cells [5].

Herpesviruses can cause periapical tissue damage through immunopathological responses, with a predominance of Th1 cells in periapical lesions, which mediate delayed-type hypersensitivity. HCMV may induce immunosuppression by reducing the expression of major histocompatibility complex class I molecules, interfering with recognition by cytotoxic T lymphocytes, while EBV may promote proliferation of cytotoxic T lymphocytes to destroy infected cells, but may also inhibit aspects of the immune response [6,7].

Several studies have been performed for both viruses, where PCR has been used to detect them in samples of apical lesions. It has been hypothesized that herpesvirus reactivation may occur spontaneously or during periods of decreased host immune defenses, which is a risk indicator for acute exacerbation of chronic apical lesions [2]. On the other hand, the presence of the Varicella-Zoster virus has also been reported in apical pathologies, although its role is unclear due to the lack of well-designed clinical studies [8].

The microorganisms present in apical lesions, which are mainly bacteria, have been extensively studied [1,5,9]. However, there is currently little knowledge about the role that viruses play in endodontic diseases, their possible effects, and the future complications that they can produce in these cases.

Fungi have also been identified within root canal infections and their contribution to periapical disease could be substantial. They are particularly prevalent in persistent root canal infections and reinfections, especially in cases where there is communication with the oral cavity or in immunocompromised individuals [10].

In addition, interactions between herpesviruses and bacteria may help to explain various clinical features of periapical infections. Interrupted periods of latency and frequent reactivation of the herpesvirus may result in disease progression and symptomatology [7].

Periapical outbreaks may develop because of complex immune responses against herpesviral–bacterial co-infection. The cumulative effects of herpesviruses, endopathogenic bacteria and proinflammatory immune mechanisms may manifest in increased periapical alveolar bone resorption and clinical symptoms such as acute pain, biting discomfort and tenderness to palpation in the apical region [4].

The objective of this review is to determine the presence of various viruses in pulp tissue and/or apical lesions and their possible roles.

## 2. Materials and Methods

The study design corresponds to a systematic review conducted according to the PRISMA reporting guidelines [11], which helps synthesize the evidence obtained on the detection of viruses in pulp and apical lesions.

Studies were searched for in the electronic databases PubMed–Medline and Web of Science (WoS) and were all published within the last 10 years. The searches were performed in PubMed using the following MeSH terms, keywords and Boolean operators: “viruses” [MeSH] and endodontic, pulpitis and virus, virus and periapical, herpesvirus and periapical, papillomavirus and periapical, Epstein Barr and periapical. On the other hand, in WoS the following keywords and Boolean operators were used: “virus” (all fields) and “endodontic” (all fields), “herpesvirus” and “periapical”, “papillomavirus” and “periapical”, “periapical” and “virus”, “pulpitis” and “virus”, “Epstein Barr” and “periapical”.

The initial evaluation of the articles was carried out by reading the title according to its relation to the topic, and duplicate articles were discarded. Subsequently, the abstract was read. Finally, the full text was read and analyzed. Articles not directly related to the inclusion criteria were immediately discarded. Additional manual searches were performed in bibliographies of previous publications, with no results.

The inclusion criteria consisted of studies published in the last 10 years (until 16 November 2023); controlled and/or randomized clinical studies, observational prevalence studies, case–control studies; studies that determined the presence of virus in pulp, apical tissue, or acute abscesses of endodontic origin, directly or indirectly; studies that specified the number of patients and/or samples, the intervention performed and the method used to determine the presence of the virus; studies published in English or Spanish.

Exclusion criteria included: studies that did not clearly indicate the type of virus present; studies that did not meet the validity criteria; studies that did not state the form or method of virus detection.

A validity assessment was performed by two of the investigators (P.S.C./K.H.H.) by reading the title and abstract and subsequently performing a complete reading of the articles to verify that they met the inclusion criteria. Disagreements between the reviewers for the inclusion of an article were resolved through a discussion among all the researchers. The articles were reviewed and verified with the Cochrane ROBINS-i tool [12].

**Figure 1:**
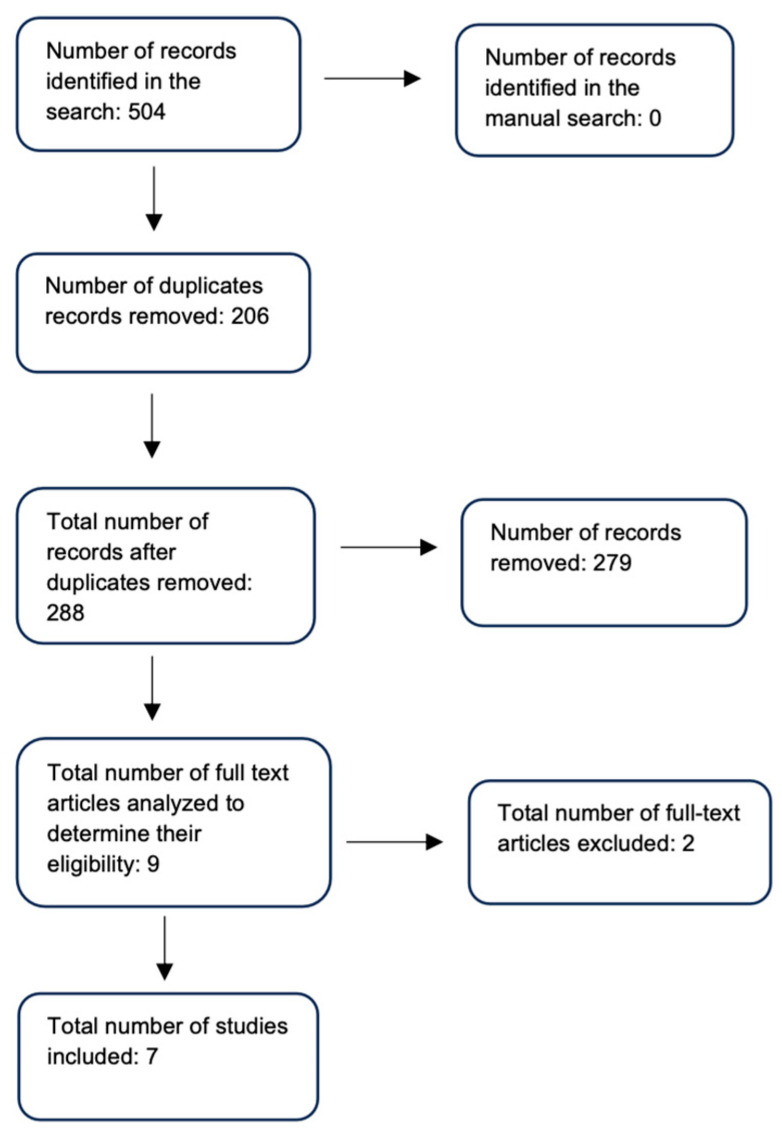
Study selection flowchart.

The study selection process is shown in Figure 1.

The nominal qualitative variables evaluated were the presence of virus, name of virus, the tissue where it was detected, the title and date the study was published, and other quantitative variables such as the percentage of samples where the presence and/or absence of the viruses studied was found.

The presence of a virus is determined by one of the following detection methods: immunohistochemistry, PCR or in situ hybridization.

The data collected were entered into an Excel spreadsheet, which contains the following information: author, year of publication, country of publication, number of samples analyzed, diagnosis, tissue analyzed, number and type of control tissue, virus detected, detection procedure and results of positive and negative samples for each publication.

**Figure 2:**
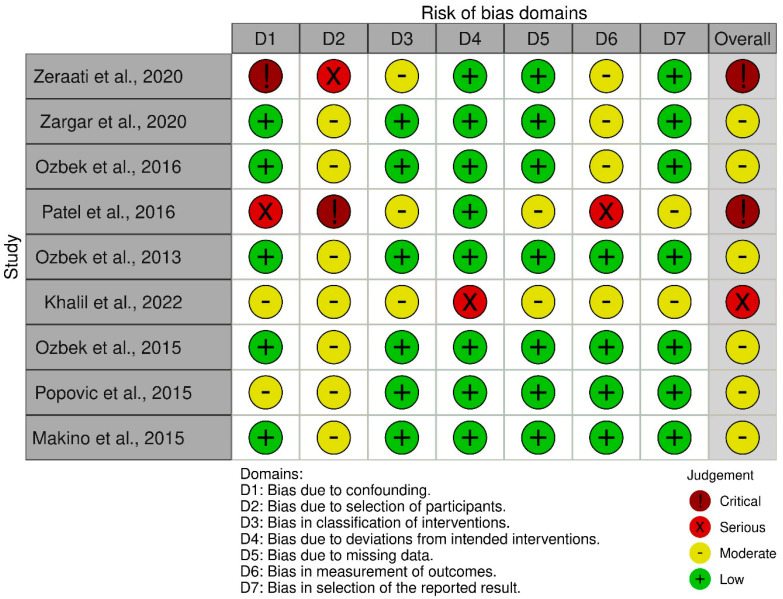
Cochrane Robins-I tool risk of bias assessment [2,5,9,14,15,16,17,18,19].

The evaluation of the risk of bias of the selected articles was carried out by applying the analysis and assessment of the 7 domains of the Cochrane ROBINS-I tool, where the two reviewers (P.S.C./K.H.H.) had this tool at their disposal independently [13]. The reviewers’ assessment was provided by judging the risk of bias as low, moderate, serious or critical. The analysis of the studies is available in Figure 2.

## 3. Results

A search for scientific evidence was performed in PubMed–Medline and Web of Science, which yielded 504 results, and manual searches were performed, which did not yield positive results. Subsequently, duplicates were eliminated, leaving a total of 288 studies. Finally, nine full-text studies were reviewed and analyzed applying the ROBINS-I guideline, where two articles were excluded because they presented a risk of critical bias. The results of the search are shown in Table 1.

Seven studies were included in this systematic review, in which the presence of different viruses in pulp tissue, periapical lesions and purulent exudates of apical abscesses were evaluated.

HCMV was tested for in four studies, with positive results in all of them. A total of 136 samples were analyzed for HCMV, which was present in 47 (34.5%).

EBV was tested for in six studies, with a total of 203 samples, 56 of which were positive (27.5%). In control tissue samples, EBV was present in a total of 23 (29.33%).

In turn, ADV was searched for in a study containing a total of 35 samples of periapical infection, of which 15 were positive (42.86%). In control tissue, ADV was positive in 21 (26.67%) of the study samples.

The presence of HPV-16 was studied in one article with a sample of 35 cases of periapical infections, where 5 were positive (14.28%). In samples of healthy control tissue, it was positive in three of them (4%).

HSV-1 was analyzed in a total of 41 samples in one study and was present in 4 of them (9.8%).

In addition, HHV-6 was searched for in 21 cases of apical abscesses, specifically purulent exudates from the affected area, appearing positive in only 1 case (4.8%).

HPV was studied in two investigations, with a total of 48 samples of apical abscesses and was present in three cases (6.25%).

HCMV and EBV were shown to be the main association between viruses, found in 19 (13.9%) out of a total of 136 samples of different diseases of endodontic origin, while the association between HCMV and HHV-6 was found in 1 sample (4.8%) out of a total of 21 cases of apical abscesses.

Details of the characteristics of the studies included in this review are presented in Table 2.

## 4. Discussion

Our findings underline the possible role of viruses in pulpal and apical diseases, which represents an approach that should be considered in endodontic treatment prognostics. This research highlights the importance of these etiological agents, which can be found in different tissues or samples of endodontic origin.

Currently, the most studied viruses in different diseases of endodontic origin are HCMV and EBV of the Herpesviridae family [2,8,14,15,16,17].

Viral infections trigger a proinflammatory response characterized by the expression of various cytokines and chemokines, which play a crucial role in the host’s immune response to infection [20].

Host defense mechanisms against viruses include the innate immune response as the first line of defense. Sensors located in the endosomal and cytosolic compartments detect viral nucleic acids, triggering the production of type I interferons and proinflammatory cytokines. These, in turn, induce interferon-stimulated genes that restrict viral infection. Although the mechanisms of viral RNA and DNA detection depend on different receptors and adaptor proteins, their downstream signaling is similar. There is a high degree of interplay between viral RNA- and DNA-sensing mechanisms in antiviral innate immunity, thus amplifying the antiviral response throughout the viral life cycle. However, critical questions remain about the overlap between these mechanisms. The interplay between them allows detection of infection throughout the viral cycle, eliminating invading pathogens and preventing damage to the host. However, viruses have evolved strategies to evade innate host immunity, suppressing this overlap [21].

Epstein–Barr virus (EBV) induces immune dysregulation by inhibiting the replication of stimulated peripheral blood mononuclear leukocytes [22]. In addition, EBV can positively regulate the production of proinflammatory cytokines, such as tumor necrosis factor-α (TNF-α), interleukin (IL)-1β, IL-6, IL-8 and IL-10. IL-10 inhibits the production of key cytokines such as IL-2 and interferon-γ (IFN-γ) by T helper type 1 (Th1) cells, which are essential for the control of viral infections [17].

Additionally, it has been suggested that EBV may be present in local inflammatory lesions, acting as a reservoir that facilitates the systemic spread of the virus [17].

Human cytomegalovirus (HCMV) infection also induces the production of proinflammatory cytokines in inflammatory cells, including IL-1β, IL-6, IL-12, TNF-α, interferon-α/β and IFN-γ [7].

Ozbek et al. 2016 reports that these herpesviruses produce an activation of inflammatory mediators, since they are potent bone resorption-stimulating agents, thus generating the hypothesis that the presence of these viruses could generate a greater loss of bone tissue in endodontic diseases [14].

Makino et al. 2015 reports that EBV is important in periapical inflammation, either in the onset or prolongation of inflammation, because of increased tissue damage. In turn, it is suggested that elevated levels of the virus in periapical granulomas help immune reactions in periapical lesions, such as tissue destruction and bone resorption. In addition, it is suggested that EBV could be present in local inflammatory lesions, as these are a reservoir of the virus that can deliver it systemically [17]. Both B cells and plasma cells present in periapical granulomas are important targets in EBV infection, as they play roles in several aspects of periapical inflammation [17].

Over the years, the Herpesviridae family has been studied in different endodontic diseases, but conclusive studies on the role of these viruses in the different clinical presentations of endodontic infections are still lacking.

The presence of viral infections in both healthy and diseased dental pulps has been studied [7,23,24]. In Li’s study, human cytomegalovirus (HCMV) was detected in healthy and inflamed pulp tissue [23]. This finding suggests that viral infections may be present even in pulp tissue that shows no obvious clinical signs of disease, underlining the need for further investigation into the role of viruses in pulp health and endodontic disease.

Viral miRNAs (vmiR), including those of herpesvirus and human cytomegalovirus, have been discovered in pulp tissue and are differentially expressed in inflamed pulp compared to normal pulp. MicroRNAs (miRs) are small single-stranded non-coding RNAs, typically 21–24 nucleotides in length, that play a crucial role in regulating the transcriptome by fine modulation of gene expression. Through this regulatory function, miRs influence a wide range of fundamental biological processes, such as cell differentiation, signaling pathways, cell death and response to pathogens. Their ability to modify these processes highlights their importance in maintaining cellular homeostasis, as well as their potential involvement in disease development and progression. Studies have shown that viruses, especially those with DNA genomes, encode miRNAs as a strategy to regulate their life cycle within the host. These viral miRs allow the virus to manipulate the host cellular processes, facilitating viral replication, persistence and evasion of the host immune response. This viral exploitation of miRs underlines their relevance in the complex interaction between pathogens and their hosts. Also, miRNAs have a possible role in processes such as cell death, viral proliferation and oncogenesis [25].

However, the specific role of viral miRNAs in endodontic disease, which would expand understanding of pulpal diseases, is not yet fully understood as historically the focus has been on bacterial infection with the possible role of emerging viral infections or co-infections. Zhong’s study reinforces the need for further research into the specific mechanisms by which viruses infect and modulate pulp tissue cells [25].

Other viruses analyzed are HHV-6, HSV-1, HPV (among them HPV-16, which is a genotype belonging to the papillomavirus family). These were less studied, and appear only in a low percentage of positive samples [5,9,14,16]. On the other hand, ADV was found in only one study [6] and presented a high percentage of positive samples in periapical infection (42.86%), while in healthy controls a positive result of 26.67% was found. The authors suggest further exploration of the subject because the prevalence of periapical infection observed was in the “low positive” category, which clinically indicates a negative result [5]. In addition, there are no previous studies on the presence of this virus in endodontic diseases.

An important aspect to highlight concerns the dual HCMV/EBV infection analyzed in a total of four studies [2,14,15,16].

In the study by Popovic et al., the sample was separated into symptomatic and asymptomatic lesions, where in symptomatic lesions, 35.48% (11 out of 31 samples) were positive for HCMV/EBV co-infection. In asymptomatic lesions (29 samples) there were no positive samples for HCMV/EBV co-infection [2].

In Ozbek’s research study in 2013, where samples were separated according to symptomatology as either symptomatic and asymptomatic, 25% of symptomatic lesions (4 samples out of 16) demonstrated positive results for dual HCMV/EBV infection [15]. However, in contrast with the results obtained by Popovic et. al, in Ozbek’s study, co-infection was found in 16.7% of asymptomatic lesions (only 2 samples out of 12) [2,15].

In another study by Ozbek et al. in 2015, 27 samples of acute apical abscesses were analyzed and dual HCMV/EBV infection was found in 1 sample (4%) [16].

The same author, in another study, analyzed the samples according to the radiographic size of periapical bone destruction in 11 large lesions (> or equal to 5 mm) and 10 small lesions (<5 mm). HCMV/EBV co-infection was found only in one large-sized sample (9%), and there were no positive results for dual infection in small-sized lesions [14].

It is presumed that HCMV and EBV could play a pathogenic role in severe cases of endodontic disease [2]. HCMV reactivation has the potential to transactivate EBV and thus create further pathogenicity of its mechanism [26]. HCMV and EBV have also been shown to exert anti-apoptotic activity. Inhibition of the apoptosis process could further result in continued inflammation and cytokine production and ultimately lead to the establishment of a chronic inflammatory stage [27].

There is no clarity on the activation of herpesviruses, which could be the cause of acute inflammation of endodontic diseases or vice versa, where it is the inflammation that finally produces this activation, which is a current research focus [2].

In relation to the symptomatology of the lesions studied, in Ozbek’s 2013 study, an attempt was made to relate HCMV, EBV and co-infection of both with clinical symptoms of symptomatic lesions of apical periodontitis, where acute pain, pain on percussion, sensitivity to palpation and swelling were found. However, the difference in the occurrence of HCMV and EBV DNA between symptomatic and asymptomatic periapical lesions was not statistically significant [15]. This presented a contrast to to the study by Popovic et al., where there was a significantly higher frequency of HCMV and EBV infection in symptomatic lesions compared to asymptomatic ones [2].

Among the effects of the viral presence in endodontic diseases is the cumulative effect caused by endodontic bacteria, which, added to the presence of the virus and the consequent destruction of the tissue mediated by the immune system, together with the general weakening of the host’s defenses, establishes a vicious circle and makes the periapical disease more aggressive [2]. This can manifest itself in increased resorption of the periapical alveolar bone and clinical symptoms such as acute pain, discomfort on biting and tenderness to palpation in the apical region of the mucosa [4].

In addition, it is noted that frequent activation of herpesvirus at the periapical level may accelerate the development of periapical defects, while the absence of herpesvirus infection or its reactivation may be the reason some periapical lesions remain clinically stable for a longer period [28].

This topic is still under study as there is no clarity on the effect of viruses on the progression and development of periapical lesions.

Of the seven studies analyzed, four presented case controls where different healthy tissues were used [5,14,16,17]; two of which [14,16] used dental pulp, while the other two studies [5,17] used gingival, alveolar and periodontal ligament tissue. The remaining three studies [2,9,15] did not present control samples, so they were not comparable with healthy tissues.

In the study by Khalil et al., the presence of viruses (ADV, EBV and HPV-16) was found in control tissues (healthy alveolar tissue and periodontal ligament). EBV presented the highest positivity in control cases with 29.33% of positive samples, followed by ADV with 26.67% and, finally, HPV-16 with 4% of positive samples in control tissue [5].

These results differ from the other studies included in this review, which presented case controls, where no presence of the studied viruses was found in the control samples [14,16,17].

One of the complications of having a greater number of samples for healthy controls is pointed out by Botero in his research, where he mentions that the number of healthy controls is limited since it is complicated to obtain healthy pulp or healthy apical periodontal tissue from teeth that have not been affected [29].

It is important to have the presence of healthy control tissues to make a true comparison of the presence of viruses and to be able to obtain more accurate results and data on whether they are only present in diseased tissue or are also found in healthy tissues. This would determine whether they really influence the development of endodontic diseases or whether they are merely a finding.

It is hoped that future studies will include healthy controls, which can be comparable both in the number of samples and type of tissues.

Arduino et. al. in a recent systematic review of HSV-1 in periapical diseases, mentions that the estimated prevalence given by PCR may overestimate the real prevalence percentage. Moreover, they did not find a causal role of HSV-1 in the development and progression of periapical disease [30]. In turn, other reviews point out that the association of HCMV with periapical periodontitis is inconclusive due to a lack of evidence [29,31].

Another virus that is important to note is SARS-CoV-2. Endodontic emergencies showed a significant increase in the post-pandemic period compared to the pre-pandemic period, suggesting a possible relationship between SARS-CoV-2 infection and exacerbation of oral diseases, particularly endodontic diseases [32].

Bacteria are traditionally the predominant etiological agents in pulpitis, co-infection with viruses, including SARS-CoV-2, and could play a critical role in the development and aggravation of these pathologies. The latent mechanisms of SARS-CoV-2 in the intensification of clinical manifestations of oral diseases are not yet fully understood, but it is hypothesized that the virus could exacerbate pre-existing periodontal and endodontic diseases or increase the severity of tissue degradation by generating new pathways of damage, leading to severe pain and increased severity of dental pathology [32].

Furthermore, persistent inflammation in periodontal and endodontic tissues, linked to SARS-CoV-2 infection, could facilitate the interaction of underlying immune mechanisms, exacerbating COVID-19-associated oral and systemic clinical symptoms through common chronic inflammatory pathways [32,33].

The study by Galicia JC et al. suggests susceptibility of dental pulp to SARS-CoV-2 infection [34], although a direct causal relationship between SARS-CoV-2 infection and endodontic disease has not been established [35]. The potential impact of this virus, especially considering its systemic effects and immunomodulatory properties, may contribute to the complexity of endodontic infections and deserves further investigation.

## 5. Conclusions

At present, the role of viruses in endodontic diseases is still unknown, as well as the effects and consequences they can produce. Advancing in this subject would help provide better treatments, focused on the different types of microorganisms found in pulpal and periapical diseases and also the presence of viruses. The greatest effect of the presence of viruses in endodontic diseases is most likely in immunocompromised patients, especially the presence of EBV and HCMV which were the most associated with endodontic disease, and which could compromise the prognosis of treatments in these patients.

Undoubtedly, further studies are required to elucidate the role of viruses, including SARS-CoV-2, in the pathogenesis and evolution of endodontic diseases.

## Figures and Tables

**Table 1:** Summary of literature search results on viruses in endodontics.

Database/Search	Number of Studies
PubMed:	
(pulpitis) and (virus)	25
(virus) and (periapical)	66
(“viruses” [MeSH]) and (endodontic)	188
(herpesvirus) and (periapical)	26
(papillomavirus) and (periapical)	7
(Epstein Barr) and (periapical)	19
Web of Science:	
“virus” and “endodontic”	61
“herpesvirus” and “periapical”	7
“papillomavirus” and “periapical”	8
“periapical” and “virus”	55
“pulpitis” and “virus”	11
“Epstein Barr” and “periapical	31
Total	504

**Table 2:** Characterization of included studies.

Author/Year/ Country	Type of Study	Number of Samples/Diagnosis/Tissues Analyzed	YES/No N° Type of Control Sample	Virus Analy-Zed	Analytical Procedure	Virus Detected	Positive Samples (%)
Ozbek S. et al. 2016, Turkey [14].	Descriptive observational	21/Apical abscesses/Purulent exudate (aspiration)	Yes, 10, healthy dental pulp (no virus-positive specimens)	HCMV, EBV, HHV-6, HPV	Real-time PCR	HCMV, EBV, HHV-6, HPV	HCMV (19%) EBV (14%) HHV-6 (4.8%) HPV (4.8%) HCMV + HHV-6 (4.8%) HCMV + EBV (4.8%)
Popovic J. et al. 2015, Serbia [2].	Descriptive observational	60/Chronic periapical lesion/Root lesion of extracted teeth	No	HCMV, EBV	PCR (qualitative pcr)	HCMV, EBV	HCMV (48.3%) EBV (21.6%) HCMV + EBV (18.3%)
Khalil W. et al. 2022, Lebanon [5].	Analytical observational	35/Periapical infections/Periodontal ligament	Yes/80/Healthy alveolar and periodontal ligament tissue	ADV, EBV, HPV-16	Real-time PCR	ADV, EBV, HPV-16	ADV (42.86%) EBV (25.71%) VPH-16 (14.28%)
Ozbek S. et al. 2013, Turkey [15].	Analytical observational	28/Apical periodontitis S and A/periapical tissue (lesion)	No	HCMV, EBV	Real-time PCR	HCMV, EBV	HCMV (32.1%) EBV (14.2%) HCMV + EBV (21.4%)
Ozbek A., Ozbek M. 2015, Germany [16].	Descriptive observational	27/Apical abscesses/Purulent exudate (aspiration)	Yes/6/Healthy dental pulps (no virus-positive samples)	HCMV, EBV, HPV	Real-time PCR	HCMV, EBV, HPV	HCMV (18.5%) EBV (7.4%) HPV (7.4%) HCMV + EBV (4%)
Makino K. et al. 2015, Japan [17].	Controlled clinical	32/Periapical granuloma/Periapical lesion	Yes, 10, Healthy gingival tissue (no virus-positive specimens)	EBV	Real-time PCR	EBV	EBV (78.1%)
Zargar N. et al. 2020, Iran [9].	Analytical observational	41/P. Irreversible and Necrosis P./Pulp	No	HSV-1	PCR	VHS	HSV-1 (9.8%)

PCR: polymerase chain reaction. HCMV: Human cytomegalovirus. EBV: Epstein–Barr Virus. HHV: Human herpesvirus, HPV: Human papillomavirus. ADV: Adenovirus. HSV: Herpes simplex virus.
